# Illinois State Veterinary Medical Association

**Published:** 1900-01

**Authors:** Albert C. Worms

**Affiliations:** Secretary


### ILLINOIS STATE VETERINARY MEDICAL ASSOCIATION.

THE seventeenth annual meeting of the Association was held at the Sherman House, Chicago, November 15 and 16, 1899.

The meeting was called to order November 15th at 11.30 A.M., President W. J. Martin in the chair. The following members responded to roll-call: Drs. W. J. Martin; Fry, of Naperville; Story, Gunning, Judson, Worms, Walker, Jas. Robertson, E. L. Quitman, J. T. Nattress, A. G. Alverson, Hugh Thompson, J. T. Ryan, J. L. Siegrosser, W. H. Welch, A. H. Baker, R. T. Hoadley, F. A. Pressler, N. I. Stringer, Albert Babb, and Brown, of Galesburg.

The minutes of the last regular meeting were approved as read, after which the meeting was adjourned.

Meeting called to order at 2.30 P.M. President Martin, in his interesting annual address, gave a résumé of the status of the veterinary profession, and called attention to the ignorance of the medical profession regarding the close connection between diseases of animals and man, and suggested that every medical college should have on its staff a veterinary instructor.

Dr. Hugh Thompson then read a paper on several interesting cases of bowel trouble, and one of apparent choke.

The same was thoroughly discussed by Drs. Nattress, Robertson, E. L. Quitman, A. H. Baker, and Alverson.

Remarks were next made by D. A. H. Baker on "The Prevailing Influenza," which was liberally discussed by Drs. Robertson, E. L Quitman, Pressler, Alverson, and Nattress.

The meeting was then adjourned until Thursday at 10 A.M., to meet at the Chicago Veterinary College for clinic.

*November* 16, 1899.

Upon reconvening at the Chicago Veterinary College the following clinics were presented :

A case of locomotor ataxia, one of fracture of the ilium just below the articulation with the sacrum. This condition was diagnosed by Dr. A. H, Baker, and the horse, which, by the way, was an old worn-out cripple, taken to the dissecting-room, where he was destroyed, and upon post-mortem examination Dr. Baker's diagnosis was confirmed.

One case of quittor: One serous cyst on hindquarter due to a kick from another horse; one suspected case of glanders. Mallein test without result.

One case of tetanus, in slings; one operation of low neurectomy, and I would state in regard to this case that healing took place by primary union in one week. Operation performed by Dr. A. H. Baker.

The clinics at the college were very instructive, and were highly appreciated by all present.

Meeting called to order at 2.30 P.M. at the Sherman House, President Martin in the chair.

Dr. Albert Babb read an interesting paper of a case of nasal polypus, the same being of extraordinary size and weighing two pounds two and one-half ounces when removed.

Operation was fully described and resulted in complete recovery. Discussed by Drs. F. H. Pressler, E. L. Quitman, A. G. Alverson, and N. I. Stringer.

Next we had a paper by Dr. James Robertson on "Our New Veterinary Law." Discussed by Drs. Nattress, Quitman, Wessler, Alverson, and Stringer.

A general discussion on tuberculosis was participated in by Drs. Martin, Robertson, Quitman, Alverson, Storey, Stringer, Babb, Ryan, and Nattress.

Dr. Ryan made a suggestion that this Association shall take action in regard to State legislation in regard to tuberculosis in this State, and said there should be something done in reference to National legislation on this subject.

The Treasurer's report was handed in, read, and on motion was approved.

The following members were elected for the ensuing term to fill the several offices: President, W. J. Martin, Kankakee; Vice-President, T. J. Gunning, Neponset; Secretary, A. C. Worms, Chicago; Treasurer, R. G. Walker, Chicago. Censors: A. H. Baker, Chicago; Albert Babb, Springfield; R. W. Story, Princeton.

After several minor discussions regarding our Constitution and By-laws, it was moved by Dr. Robertson, seconded by Dr. Siegrosser, that a vote of thanks be extended to Dr. A. H. Baker for the clinics held at the Chicago Veterinary College.

A vote of thanks was tendered Drs. Walker, Ryan, and Robertson for the clinics which they furnished. A vote of thanks was extended to J. Irving Pearce, proprietor of the Sherman House, for his kindness and hospitality to the veterinary profession.

Motion made and carried that a vote of thanks be extended to Messrs. E. R. Squibb, Schering, and Glatz, and Merck & Company for the splendid display of drugs and chemicals exhibited at the Association meeting.

It was moved and seconded that our next semi-annual meeting be held at Springfield, Ill.; carried.

Motion made to adjourn, which was carried.

ALBERT C. WORMS,
Secretary.

